# High-Fat/High-Sugar Diet and High-Temperature/High-Humidity Exposure Aggravates Ulcerative Colitis in an Experimental Mouse Model

**DOI:** 10.3390/cimb47070562

**Published:** 2025-07-18

**Authors:** Pengyan Li, Guibing Meng, Ang Li, Liang Chen, Xinchi Feng, Feng Qiu

**Affiliations:** 1 Tianjin Key Laboratory of Therapeutic Substance of Traditional and State Key Laboratory of Component-Based Chinese Medicine, School of Chinese Materia Medica, Tianjin University of Traditional Chinese Medicine, Tianjin 301617, China; pengyanlpy@163.com (P.L.); mgb901029@163.com (G.M.); lianghbkd@163.com (A.L.); 2 School of Pharmacy, Guizhou University of Traditional Chinese Medicine, Guiyang 550025, China; chenliang029@gzy.edu.cn

**Keywords:** ulcerative colitis, high-fat/high-sugar diet, high-temperature/high-humidity exposure, inflammation, intestinal permeability

## Abstract

Ulcerative colitis (UC) is a subtype of inflammatory bowel disease (IBD) that has been associated with overconsumption of calories and lipids, compared to the healthy population, and summer temperatures have been reported to be closely related to the prevalence of UC. To evaluate the effects of dietary and lifestyle factors on UC, a combination of 2.0% dextran sulfate sodium (DSS), a high-fat/high-sugar diet, and exposure to high temperature and humidity was used to construct mouse models of UC. Changes in body weight, disease activity index (DAI) scores, histopathological analysis, serum lipid levels, serum diamine oxidase (DAO), and D-Lactate (D-LA) levels, as well as the expression of inflammatory cytokines and tight junction proteins in colonic tissue, were all assessed to study the impacts of the high-fat/high-sugar diet and high-temperature/high-humidity exposure on the progression of UC. The symptoms observed in the UC mouse model induced by 2.0% DSS alone were similar to those seen in patients with UC, while the high-fat and high-sugar diet, along with humid and hot exposure, exacerbated DSS-induced UC in the mice. This included more severe histopathological damage to the colon tissue, increased expression of pro-inflammatory cytokines (IL-6, IL-17A, and IL-1β), and a more significantly compromised intestinal barrier, characterized by the destruction of ZO-1 and elevated levels of DAO and D-LA. Additionally, the high-fat/high-sugar diet and high-temperature/high-humidity exposure led to further disturbances in glucose and lipid metabolism in the mice, which were not observed in those treated with DSS alone. This study is the first to investigate the effects of a high-fat/high-sugar diet and high-temperature/high-humidity exposure on the progression of UC.

## 1. Introduction

Ulcerative colitis (UC) is a chronic inflammatory bowel disease (IBD) that primarily affects the colon. It is clinically characterized by symptoms such as diarrhea, mucus and purulent blood in the stool, and abdominal pain [1,2]. Recently, the incidence and prevalence of UC have gradually increased worldwide [3]. Although the pathogenesis of UC is not fully understood, it is widely accepted that various factors may contribute to the progression of this disease, including exogenous summer heat and dampness, improper diet, and the consumption of contaminated or spoiled food [4]. Among these factors, dietary habits and summer climate are considered particularly significant. Dietary factors associated with an increased incidence and progression of IBD include a high intake of total fats, processed foods, and refined sugars, along with a low intake of vegetables, fruits, and dietary fiber. Dietary fiber may help maintain the integrity of the gut epithelial barrier and reduce inflammation, often through interactions with the gut microbiota. These interactions appear to play a significant role in the inflammatory mechanisms within the gut, as well as in the incidence and progression of IBD [5,6]. Additionally, summer temperatures have been shown to have a significant correlation with the prevalence of UC [7].

As for UC, dextran sodium sulfate (DSS) is the most commonly used chemical inducer for UC animal models due to its low cost, simplicity, stability, and reliability [8]. Various factors, including diet, lifestyle, and summer climate, can influence the development of UC. In addition to DSS, other methods such as high-fat and high-sugar diets, exposure to high temperatures and humidity, honey-sweetened drinking water, alcohol treatment, and abnormal patterns of starvation and refeeding have all been employed to induce UC in animal models [9,10,11,12]. Among these induction methods, high-fat and high-sugar diets, as well as high-temperature and humidity exposure, are the most frequently utilized. Although researchers believe that these stimuli can replicate the lifestyle habits of UC patients, the specific effects of these stimuli on the pathological symptoms of UC in animal models have yet to be fully elucidated.

To investigate the effects of dietary and lifestyle habits on the pathological progression of UC, we employed a UC model induced by DSS, combined with factors such as a high-fat and high-sugar diet, as well as exposure to humid and hot conditions, to assess their impact on the UC model. We systematically compared pathological changes, including the general health status, blood lipid and glucose levels, inflammatory markers, and intestinal permeability, with the results observed in mice models of UC induced solely by DSS treatment. The aim was to elucidate the influence of diet and environmental factors on UC models. Furthermore, the UC mouse model established and characterized in this study can be utilized for research into the pathogenesis of UC and the development of potential therapeutics.

## 2. Material and Methods

### 2.1. Reagents

Dextran sulfate sodium (DSS, molecular weight of 36–50 kDa, Cat#216011090) was purchased from MP Biomedicals (Santa Ana, CA, USA). A high-fat and high-sugar diet was purchased from Xiao Shu You Tai Biotechnology Co., Ltd. (Beijing, China), and the energy supply ratio was 67.4% basal feed + 15% sucrose + 10% lard + 10% yolk powder + 4% cholesterol + 0.03% sodium cholate. The total cholesterol (TC) assay kit (Cat#A111-1-1), triglyceride (TG) assay kit (Cat#A110-1-1), high-density lipoprotein cholesterol (HDL-C) assay kit (Cat#A112-1-1), low-density lipoprotein cholesterol (LDL-C) assay kit (Cat#A113-1-1), glucose (GLU) assay kit (Cat#A154-1-1), and myeloperoxidase (MPO) assay kit were purchased from Nanjing Jiancheng Bioengineering Institute (Nanjing, China). The diamine oxidase (DAO) test kit (Cat#ADS-W-YH007-96) was purchased from Jiangsu Meimian Industrial Co., Ltd. (Yancheng, China). The D-lactate (D-LA) test kit (Cat#ADS-F-T017) was purchased from Jiangsu Aidisheng Biological Technology Co., Ltd. (Yancheng, China). The primary antibody against ZO-1 (R40.76, Cat#sc-33725) was purchased from Santa Cruz Biotechnology Inc. (Santa Cruz, CA, USA). Primary antibodies against claudin-2 (E1H9O, Cat#48120), occludin (E6B4R, Cat#91131s), and β-actin (13E, Cat#4970T) were purchased from Cell Signaling Technology, Inc. (Beverly, MA, USA). Goat anti-rat IgG H&L antibody (Cat#abs20199) and goat anti-rabbit IgG H&L (Cat#abs20040) were purchased from absin Biotechnology Co., Ltd. (Shanghai, China).

### 2.2. Animals

C57BL/6J mice (specific pathogen free grade, 6 weeks old) were purchased from SPF Biotechnology Co., Ltd. (Beijing, China). All mice were housed in a controlled environment (temperature, 25 ± 1 °C; humidity, 50 ± 5%; 12 h light/12 h dark cycle) with unlimited access to food and water. After acclimatization for one week, the mice were randomly divided into the control group, the high-fat/high-sugar diet and high-temperature/high-humidity exposure (DH) + 2.0% DSS group, and the 2.0% DSS group (*n* = 12 per group). Animal experiments were performed with the permission of Tianjin University of Traditional Chinese Medicine’s Animal Care and Use Committee (Tianjin, China; Permit No. TCM-LAEC 2022204), according to the guidance of “Guide for the Care and Use of Laboratory Animals”.

### 2.3. Experiment Design and Sample Collection

**Figure 1:**
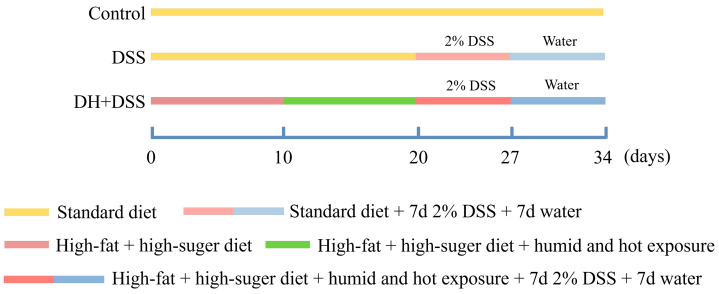
The process of experiment design. DSS: dextran sulfate sodium; DH + DSS: high-fat and high-sugar diet and humidity and hot exposure + 2% DSS; d: day.

Figure 1 shows the detailed process of the experiment design. Both a high-fat and high-sugar diet and a high temperature and humidity environment were applied to mimic the improper eating and lifestyle habits of patients with UC [5]. Mice in the control group were given a standard diet and housed in a normal environment (temperature, 25 ± 1 °C; humidity, 50 ± 5%). Mice in the 2.0% DSS group were fed with a standard diet and housed in a normal environment (temperature, 25 ± 1 °C; humidity, 50 ± 5%), and from day 20 to day 27, mice in 2.0% DSS group were fed with drinking water containing 2.0% DSS. As for the mice in DH + 2.0% DSS group, they were fed with a high-fat and high-sugar diet from day 1 to day 27, and for the first ten days, they were housed in a normal environment (temperature, 25 ± 1 °C; humidity, 50 ± 5%). From day 10 to day 20, mice in the DH + 2.0% DSS group were subjected to a 10 h humid and hot exposure (temperature, 35 ± 1 °C; humidity, 90 ± 2%) in a hot chamber (Beijing Luxi Technology Co., Ltd., Beijing, China, DWX-400FP) once daily. From day 20 to day 27, mice in the DH + 2.0% DSS group were fed with drinking water containing 2.0% DSS. Finally, all the mice were fed for another seven days to allow the progression of the disease to continue.

At the end of the experiment, all the mice were anesthetized by intraperitoneally administration of tribromoethanol (400 mg/kg). Then, serum samples were collected via cervical dislocation to determine biochemical indicators. After the collection of serum, all the mice were sacrificed, and the length values of the colon were measured. Then, the colons were divided into two parts: one was fixed in 4% paraformaldehyde, and the other was snap frozen in liquid nitrogen for future analysis. Serum and frozen colon samples were stored at −80 °C. The spleen was weighed, and the spleen index was calculated. Index of spleen = wet weight of spleen (mg)/body weight (g).

### 2.4. Disease Activity Index (DAI) Score

All mice were checked daily to monitor the state of disease progression until the end of the experiment. The body weight, stool consistency, and the presence of blood in stool for each mouse were recorded daily. The Disease Activity Index (DAI) was calculated based on the previous literature (DAI evaluation criteria was presented in Table S1) [13]. The body weight loss was scored using the following scales: 0 for no loss, 1 for a 0–5% loss, 2 for a 6–10% loss, 3 for an 11–20% loss, and 4 for a weight loss greater than 20%. The hematochezia scores were calculated as follows: 0, no blood detected by fecal occult blood test analysis; 2, a positive fecal occult blood test; and 4, extensive bleeding. For stool, 0 represented well-formed pellets, 2 represented semi-formed stools, and 4 represented liquid stools adhering to the anus. DAI score value = (score for weight loss + sore for stool property + score for the presence of blood stool)/3.

### 2.5. Histological Analysis and Serum Biochemical Indicator Detection

After being fixed in 4% paraformaldehyde for a week, the colonic tissues were embedded in paraffin. Then, the embedded tissues were cut into 5 μm sections, and H&E staining and AB-PAS staining were conducted for histological analysis. The histopathological changes were recorded, the extent of colonic tissue damage, including the intestinal epithelial cell damage and inflammatory infiltration, were quantitatively evaluated, and the specific pathology scoring criteria were briefly described as follows: 0 points for normal morphology and no inflammation; 1 point for slight inflammatory infiltration; 2 points for crypt glands and epithelium damage and moderate inflammatory infiltration of the mucosal layer; 3 points for partial loss of crypt (complete absence of crypts) and extensive inflammatory infiltration of the mucosal muscle layer with mucosal edema and thickening; and 4 points for extensive loss of crypt (complete absence of crypts) and extensive inflammatory infiltration of the submucosal layer; all the above inflammatory cell infiltration primarily refers to the accumulation of lymphocytes and plasma cells [14].

Serum TG, TC, HDL-C, LDL-C, GLU, and MPO levels were measured according to the instructions in the commercial kits.

### 2.6. Reverse Transcription Polymerase Chain Reaction (RT-qPCR)

Total RNA was extracted from the colonic tissues using Trizol reagent (R1100, Solarbio, Beijing, China). After the integrity and concentration of the extracted RNA were measured, the total RNA was reverse transcribed to cDNA with an S1000TM Thermal Cycler by using StarScript III RT kit (A232-02, GenStar^®^, Beijing, China). qRT-PCR was performed using 2 × RealStatFast SYBR qPCR Mix (A302-05, GenStar^®^). The program of PCR amplification was set as follows: 1 cycle of 95 °C for 2 min, followed by 40 cycles of 95 °C for 10 s and 60 °C for 60 s. Table S2 shows the primer sequences used in this study.

### 2.7. Western Blotting Analysis

Colonic tissues were homogenized with RIPA lysis buffer (P0013C, Beyotime, Shanghai, China) containing protease inhibitor. The protein concentrations were measured using a BCA assay kit (P0010, Beyotime). Then, 20 μg protein for each sample were separated by SDS-PAGE gel and transferred to a nitrocellulose membrane. After blocking with 5% (*w*/*v*) skim milk for 2 h, the membranes were incubated with the respective primary-antibodies overnight (4 °C). Next, the membranes were incubated with the corresponding secondary antibodies at room temperature for 2 h. Finally, bands were detected with an Amersham Imager 600 (GE Healthcare Bio-Sciences AB, Danderyd, Sweden), and the quantification of bands was conducted with Image J (v1.8.0).

### 2.8. Statistical Analysis

Statistical analysis was performed using GraphPad Prism 9.5 software (GraphPad Software, San Diego, CA, USA). Statistical differences were analyzed using one-way analysis of variance (ANOVA). Data were presented as the mean ± standard error of the mean (SEM). Values of *p* less than 0.05 were considered to be statistically significant.

## 3. Results

### 3.1. General Assessment of Mice in Different Groups

**Figure 2:**
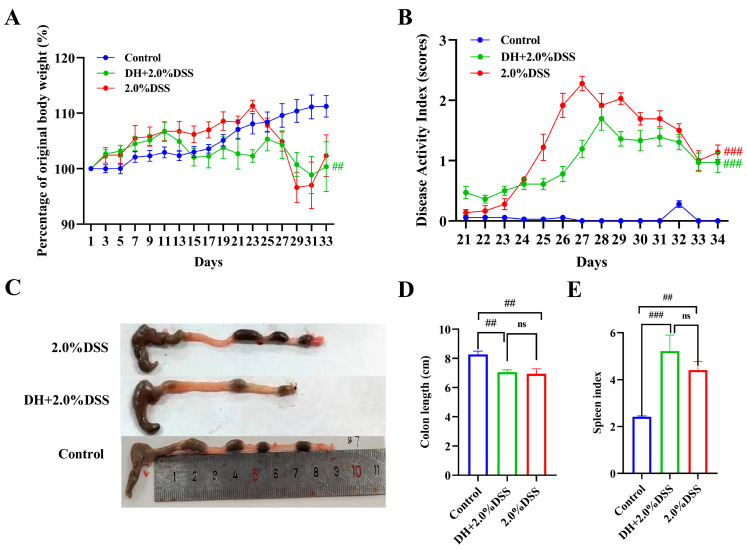
General conditions of mice in different groups. (**A**) Body weight changes; (**B**) Disease Activity Index (DAI) scores; (**C**) representative pictures of colon tissues; (**D**) colon length; (**E**) spleen index. All data are expressed as the mean ± SEM, and *p* > 0.05 displayed as “ns”. ^##^ *p* < 0.01, ^###^ *p* < 0.001 vs. the control group.

After being treated with 2% DSS, all 12 mice in both the 2% DSS group and the DH + 2% DSS group exhibited the characteristics of UC. Figure 2 shows the results of the body weight changes, DAI scores, colon length, and spleen index of mice in different groups. As shown in Figure 2A, the body weight of mice in the control group increased steadily during the whole study. As for the mice in the 2.0% DSS group, they were given 2.0% DSS from day 20 to 27, and a severe body weight loss was observed at day 23. As for the mice in the DH + 2.0% DSS group, body weight loss was observed from day 11, since from day 10, the mice were subjected to a 10 h humid and hot exposure once daily. The DAI scores of mice in the different groups were calculated from day 21 (the day administration of DSS started), and the results are displayed in Figure 2B. The DAI scores in the 2% DSS group and the DH + 2.0% DSS group initially increased until they began to decrease on days 27 and 28, respectively. However, on day 34, the scores increased significantly compared to the control group (*p* < 0.001). Furthermore, the colon length values of the mice in the 2.0% DSS and DH + 2.0% DSS groups were notably shorter than that of the control group (*p* < 0.01); however, there was no significant difference between these two groups (Figure 2C). At the end of the animal experiment, the spleen index values of mice in the different groups were calculated (Figure 2D). Compared with the control group, the values of the spleen index in the 2.0% DSS and DH + 2.0% DSS groups significantly increased (*p* < 0.01 and *p* < 0.001, respectively), indicating that splenomegaly occurred. Taken together, a high-fat and high-sugar diet along with humid and hot exposure significantly disturbed the physiological state of mice and caused obvious body weight loss and serious UC pathological symptoms.

### 3.2. Histopathological Changes

**Figure 3:**
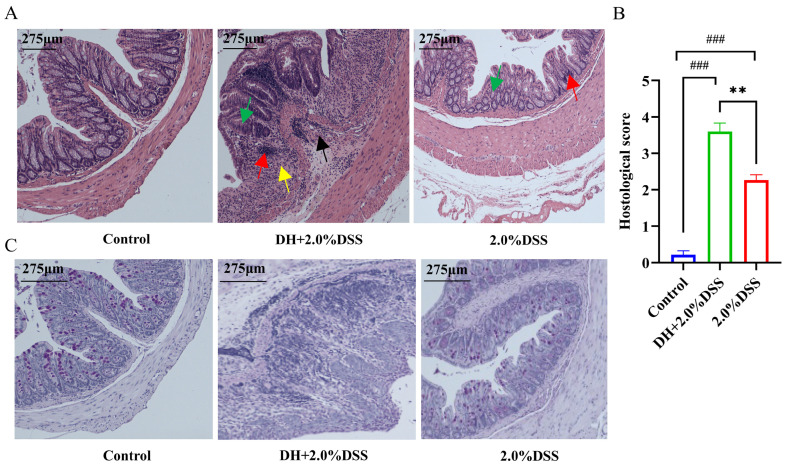
Histopathological analysis of colonic tissues in each group. (**A**) Representative results hematoxylin and eosin (H&E) staining. Red arrows indicate inflammatory cell infiltration; yellow arrows indicate plasma cells; black arrows indicate crypt abscess; green arrows indicate loss of crypt structure and depletion of goblet cells. (**B**) Pathological lesion score of colonic tissues in each group. (**C**) Representative results of Alcian blue-periodic Acid-Schiff (AB-PAS) pathological staining. The images captured at an original magnification of 10x are displayed with a scale of 175 μm. Data are expressed as the mean ± SEM. ^###^ *p* < 0.001 vs. the control group; ** *p* < 0.01 vs. the 2.0% DSS group.

Both hematoxylin and eosin (H&E) and Alcian blue-periodic Acid-Schiff (AB-PAS) staining were conducted to further analyze the histopathological changes in mice across different groups. There were no histological changes in the control group, and colonic crypts distortion, goblet cell depletion, and inflammatory cells infiltration were observed in the 2.0% DSS and DH + 2.0% DSS groups. Furthermore, the histopathological scores of colonic tissues in the DH + 2.0% DSS groups were significantly higher than that in the 2.0% DSS group (Figure 3B, *p* < 0.01). Additionally, the observation of goblet cell reduction and the colonic epithelial mucosa thickness thinning, indicated that the high-fat and high-sugar diet along with humid and hot exposure could damage the colonic mucosal barrier.

### 3.3. Serum Biochemical Profiles and Colonic Tissue Myeloperoxidase (MPO) Activity

**Figure 4:**
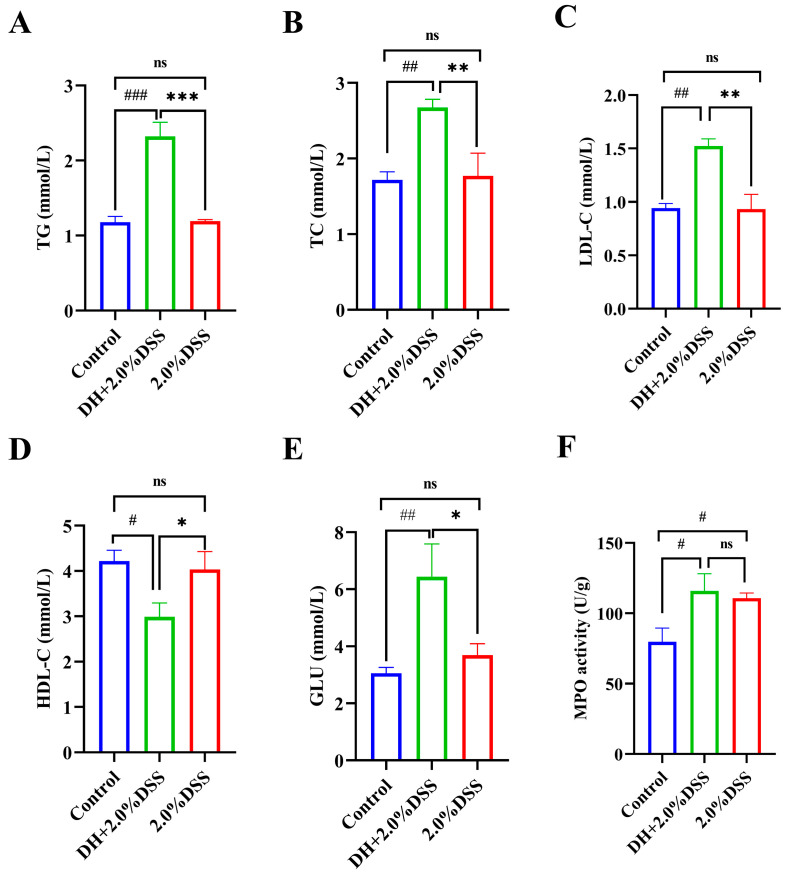
Serum biochemical profiles and colonic tissue myeloperoxidase (MPO) activity. (**A**) Serum triglyceride (TG) level; (**B**) serum total cholesterol (TC) level; (**C**) serum low-density lipoprotein cholesterol (LDL-C) level; (**D**) serum high-density lipoprotein cholesterol (HDL-C) level; (**E**) serum glucose (GLU) level; (**F**) colonic tissue MPO activities. All data are expressed as the mean ± SEM, and *p* > 0.05 displayed as “ns”. ^#^ *p* < 0.05, ^##^ *p* < 0.01, ^###^ *p* < 0.001 vs. the control group; * *p* < 0.05, ** *p* < 0.01, *** *p* < 0.001 vs. the 2.0% DSS group.

Since a high-fat and high-sugar diet was given to the mice in the DH + 2.0% DSS group, the changes in the serum lipid and glucose levels were determined. As shown in Figure 4A–E, compared with the control group, there were no significant differences in the serum levels of triglyceride (TG), total cholesterol (TC), low-density lipoprotein cholesterol (LDL-C), high-density lipoprotein cholesterol (HDL-C), and glucose (GLU) of mice in the 2.0% DSS group. However, for mice in the DH + 2.0% DSS group, the levels of TG, TC, and LDL-C were significantly increased (*p* < 0.001, *p* < 0.01, and *p* < 0.01, respectively), and the level of HDL-C was significantly decreased (*p* < 0.05) when compared with the control group. Moreover, the glucose levels of mice in the DH + 2.0% DSS group were significantly higher than that in the control group (*p* < 0.001). In summary, a high-fat and high-sugar diet along with humid and hot exposure treatment significantly caused glucose and lipid metabolism disorder.

The colonic tissue MPO activity was measured, and the results are displayed in Figure 4F. Compared with the control group, the levels of colonic tissue MPO activity in both the 2.0% DSS and DH + 2.0% DSS groups were significantly increased (*p* < 0.05). However, there was no significant difference between the 2.0% DSS and DH + 2.0% DSS groups (Figure 4F).

### 3.4. Colonic Tissues’ Inflammatory Cytokines Expression

**Figure 5:**
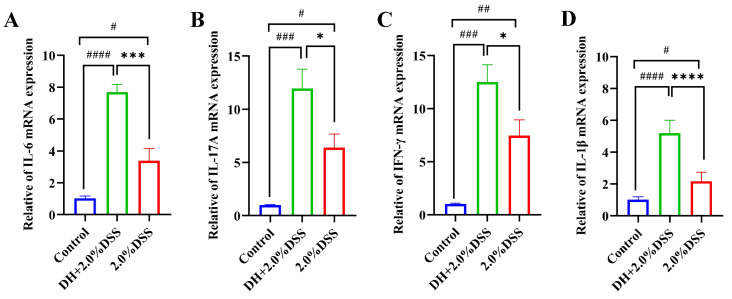
The expression levels of IL-6 (**A**), IL-17A (**B**), IFN-γ, (**C**) and IL-1β (**D**) in colonic tissues by using RT-qPCR. All data are expressed as the mean ± SEM. ^#^ *p* < 0.05, ^##^ *p* < 0.01, ^###^ *p* < 0.001, ^####^ *p* < 0.0001 vs. the control group; * *p* < 0.05, *** *p* < 0.001, **** *p* < 0.0001 vs. the 2.0% DSS group.

The results of the RT-qPCR analysis of colonic tissue inflammatory cytokines are presented in Figure 5. Compared to the control group, the expression levels of IL-6, IL-17A, IFN-γ, and IL-1β were significantly increased in both the 2.0% DSS and the DH + 2.0% DSS groups. Furthermore, the expression levels of IL-17A, IFN-γ, and IL-6 in the DH + 2.0% DSS group were statistically higher than that in the 2.0% DSS group, indicating that a high-fat and high-sugar diet along with humid and hot exposure can cause more severe inflammation.

### 3.5. Changes in Intestinal Permeability

**Figure 6:**
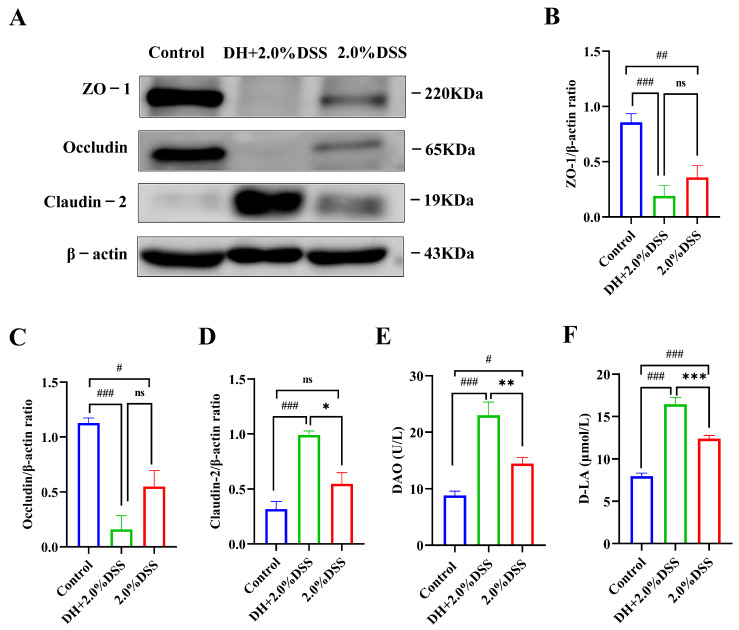
Changes in intestinal permeability. (**A**–**D**) The Western blotting results of ZO-1, occludin, and claudin-2 in colonic tissues; (**E**) the serum level of diamine oxidase (DAO); (**F**) the serum level of D-lactate (D-LA). All data are expressed as the mean ± SEM, and *p* > 0.05 displayed as “ns”. ^#^ *p* < 0.05, ^##^ *p* < 0.01, ^###^ *p* < 0.001 vs. the control group; * *p* < 0.05, ** *p* < 0.01, *** *p* < 0.001 vs. the 2.0% DSS group.

The expression levels of colonic tissue tight junction proteins, including ZO-1, oc-cludin, and claudin-2, were analyzed to evaluate the intestine barrier function using the Western blotting method, and the results are presented in Figure 6A–D. Compared with the control group, 2.0% DSS treatment could decrease the expression of ZO-1 and occludin, as well as increase the expression of claudin-2. As for ZO-1 and occludin, a high-fat and high-sugar diet along with humid and hot exposure could further decrease their expression, even though no statistical differences were observed. However, for claudin-2, the expression levels in the DH + 2.0% DSS group were significantly increased when compared with that in the 2.0% DSS group (*p* < 0.05).

Under normal physiological conditions, the serum concentrations of diamine oxidase (DAO) and d-lactate (D-LA) were very low. However, when intestinal epithelial cell injury occurred, DAO in the intestinal mucosa and D-LA in the intestinal cavity could enter the blood through the damaged site. Therefore, serum levels of DAO and D-LA have been demonstrated to be a reliable indicator of intestinal barrier injury [15]. As shown by the results in Figure 6E,F, 2.0% DSS treatment significantly increased the serum level of DAO and D-LA (*p* < 0.05, *p* < 0.001 vs. the control group). Nevertheless, the serum levels of DAO and D-LA in DH + 2.0%DSS groups were remarkably increased (*p* < 0.001 vs. the control group), indicating that a high-fat and high-sugar diet along with humid and hot exposure treatment caused more serious intestinal epithelial cell damage, which was consistent with the results of tight junction protein expression.

## 4. Discussion

In China, the prevalence of UC was estimated to be 11.6 per 100,000 individuals in 2016 [16]. The occurrence and progression of UC are influenced by various factors, including dietary and environmental influences, genetic susceptibility, lifestyle choices, and alterations in intestinal flora; however, its exact cause remains unclear [17]. Studies have found that lifestyle factors such as smoking, physical inactivity, an unhealthy diet, and abnormal sleep duration are positively associated with an increased risk of UC [18,19]. Both genetic and lifestyle factors have been independently linked to susceptibility to incident IBD. Adherence to a favorable lifestyle was associated with nearly a 50% lower risk of IBD among participants at high genetic risk [18]. Additionally, alcohol-induced reductions in Bifidobacteria and increased endotoxin production have been suggested to exacerbate intestinal inflammation in patients with IBD [20].

In addition to the aforementioned factors, dietary influences emerged as the most significant. Reports indicate that the proliferation of the “Western” diet—characterized by high levels of fat and protein but low in fruits, vegetables, and fiber—may be linked to an increased incidence of IBD [21]. Dietary fiber may play a crucial role in maintaining the gut epithelial barrier and reducing inflammation, often through interactions with the gut microbiota [6]. A high-fat diet has long been recognized as a risk factor for the progression of UC, and it has been reported that it exacerbated colonic inflammation by promoting ferroptosis [22]. Studies have shown that summer heat and dampness have a certain impact on the process of UC and affect the host’s immune function and species richness of the gut microbiome [7]. Thus, the impacts of a high-fat/high-sugar diet and high-temperature/high-humidity exposure on the progression of UC were evaluated in this study. The results showed that a high-fat and high-sugar diet and high-temperature and high-humidity exposure aggravated UC in an experimental mouse model. Specially, a high-fat/high-sugar diet and high-temperature/high-humidity exposure caused serious glucose and lipid metabolism disorder.

UC is characterized by an inflammatory response, and the imbalance between pro-inflammatory and anti-inflammatory cytokines plays a key role [23]. This imbalance can cause a disruption of the normal functioning of the intestinal barrier, causing an increase in intestinal permeability [24]. In this study, the pathology observed in mice models (both the 2.0% DSS group and DH + 2.0% DSS group) such as weight loss, hemorrhagic diarrhea, and neutrophilic infiltration, was similar to that observed in UC patients. MPO is an enzyme expressed mainly in neutrophils, and the increase in its activity has been demonstrated to be a reliable indicator of inflammation [25]. The levels of colonic tissue MPO activity in both the 2.0% DSS and DH + 2.0% DSS groups were significantly increased. Additionally, mice in the DH + 2.0% DSS group showed a more serious inflammatory response, indicating that a high-fat and high-sugar diet and high-temperature and high-humidity exposure can promote the occurrence of UC and further increase the degree of inflammation.

The intestinal barrier’s dysfunction is another key factor during the development of UC [26]. An intact barrier could effectively prevent harmful substances and pathogens from penetrating into the body, while a damaged barrier allows for their entry, leading to intestinal inflammation [27]. DAO and D-LA have been proved to be chemical indicators of intestinal permeability, which indirectly reflect the integrity of the gut [28]. DAO is an enzyme located within the villus epithelial cells of the intestine, and D-LA is an intestinal flora-derived metabolite [29]. DAO and D-LA could be released into blood circulation once the intestinal barrier is impaired [30]. In this study, serum levels of DAO were increased in the 2.0% DSS and DH + 2.0% DSS groups compared with the control group, indicating that the intestinal barrier was damaged. Mice in the DH + 2.0% DSS group showed a significant increase in the level of D-LA, suggesting that a high-fat/high-sugar diet and high-temperature/high-humidity exposure can further induce the damage of intestinal barrier, which was also proved by the results of the intestinal tight junction proteins expression.

The effects of different concentrations of DSS (1.0%, 1.5%, 2.0%, 2.5%, and 3.0%) on the establishment of UC models were also investigated. According to the results of the DAI scores, the colonic histopathology, colon length, and spleen index, with the increase in the DSS concentration (1.0% to 3.0%), the symptoms of UC gradually increased (Figure S1). In terms of the colon length and spleen index, when compared with the control group, no statistical difference was observed in the 1.0% and 1.5% DSS groups, indicating that the concentration of DSS used in this study should be higher than 1.5%. The mortality rate is another factor should be considered during the establishment of animal models. Gu et al. found that the mortality rate of a 3.0% DSS-induced mouse model of UC was 30%, whereas the mortality rate of a 2.5% DSS-induced mouse model was 10% [31]. Likewise, Wu et al. reported that a 2.5% DSS-induced mouse model of UC had a mortality rate of approximately 30% [32]. In our study, no deaths of mice were observed when 2.0% DSS combined with a high-fat and high-sugar diet and humid and hot exposure were applied to establish the mice model. Thus, an additional experiment was conducted to evaluate the mortality rate of models induced with a high-fat and high-sugar diet and humid and hot exposure combined with 3.0% DSS. The results showed that the mortality rate of mice in the DH + 3.0% DSS group was approximately 60%, which was similar to what was previously reported (Figure S2). Taken together, the combination used of DSS (2.0% or 2.5%), a high-fat and high-sugar diet, and humid and hot exposure was recommended for the construction of a UC mouse model.

However, this study has some limitations. UC is a chronic and recurrent inflammatory disease, and establishing a model induced by multiple doses of DSS may better align with the pathogenesis of UC. The high-fat/high-sugar diet and exposure to high temperature and humidity in this study are independent factors affecting UC. Creating a group that experiences both a high-fat/high-sugar diet and high-temperature/high-humidity exposure may provide a more accurate representation of UC’s characteristics. Additionally, alterations in the intestinal flora play a significant role in the progression of UC, and the effects of a high-fat/high-sugar diet and high-temperature/high-humidity exposure on the intestinal flora should also be investigated. In summary, our results showed that high-fat and high-sugar diet along with humid and hot exposure significantly aggravated DSS-induced mice UC. Additionally, no glucose and lipid metabolism disorder were observed in DSS-induced UC model, however, after introducing a high-fat and high-sugar diet and high-temperature and high-humidity factors in our study, the UC models showed significant glucose and lipid metabolism disorder. It has been reported that patients with IBD suffered a two times higher risk of developing non-alcoholic steatohepatitis compared with healthy subjects, and the abnormal lipid metabolism was thought to be one of the key reasons [33]. Additionally, various cholesterol biosynthesis genes were significantly upregulated in UC patients [34]. Taken together, a high-fat and high-sugar diet and high-temperature and high-humidity factors also affect lipid metabolism in UC mice.

## 5. Conclusions

In summary, a high-fat and high-sugar diet along with humid and hot exposure in our study could reflect the dietary and living climate characteristics of UC patients. They aggravated UC in DSS-induced mice and caused serious glucose and lipid metabolism disorders, which was closer to the clinical symptoms of UC patients.

## Data Availability

All data are provided in the manuscript.

## Supplementary Materials

The following supporting information can be downloaded at: https://www.mdpi.com/article/10.3390/cimb47070562/s1.

## Abbreviations

AB-PAS, Alcian blue-periodic Acid-Schiff; ANOVA, analysis of variance; CHO, total cholesterol; DAI, Disease Activity Index; DAO, serum diamine oxidase; DH, high-fat/high-sugar diet and high-temperature/high-humidity exposure; D-LA, D-Lactate; DSS, dextran sulfate sodium; HDL-C, high-density lipoprotein cholesterol; H&E, hematoxylin and eosin; IBD, inflammatory bowel disease; LDL-C, low-density lipoprotein cholesterol; MPO, myeloperoxidase; RT-qPCR, reverse transcription polymerase chain reaction; SEM, standard error of the mean; T-CHO, total cholesterol; TG, triglycerides; T-GLU, glucose; UC, Ulcerative colitis.

## References

[1] Li M.Y., Li M.X., Xu N., Li Z.H., Zhang Y.M., Gan Y.X., Luo H.J., Zhou C.L., Liu Y.H., Su Z.R. (2021). Effects of Huangqin Decoction on ulcerative colitis by targeting estrogen receptor alpha and ameliorating endothelial dysfunction based on system pharmacology. J. Ethnopharmacol..

[2] Neurath M. (2017). Current and emerging therapeutic targets for IBD. Nat. Rev. Gastroenterol. Hepatol..

[3] Kaplan G.G., Ng S.C. (2016). Globalisation of inflammatory bowel disease: Perspectives from the evolution of inflammatory bowel disease in the UK and China. Lancet Gastroenterol. Hepatol..

[4] Yao W., Yang C., Wen Y., Zhang W., Zhang X., Ma Q., Ji P., Hua Y., Wei Y. (2017). Treatment effects and mechanisms of Yujin Powder on rat model of large intestine dampness-heat syndrome. J. Ethnopharmacol..

[5] Principi M., Losurdo G., Iannone A., Contaldo A., Deflorio V., Ranaldo N., Pisani A., Ierardi E., di Leo A., Barone M. (2018). Differences in dietary habits between patients with inflammatory bowel disease in clinical remission and a healthy population. Ann. Gastroenterol..

[6] Christensen C., Knudsen A., Arnesen E.K., Hatlebakk J.G., Sletten I.S., Fadnes L.T. (2024). Diet, Food, and Nutritional Exposures and Inflammatory Bowel Disease or Progression of Disease: An Umbrella Review. Adv. Nutr..

[7] Aamodt G., Bengtson M.B., Vatn M.H. (2013). Can temperature explain the latitudinal gradient of ulcerative colitis? Cohort of Norway. BMC Public Health.

[8] Li Q., Cui Y., Xu B., Wang Y., Lv F., Li Z., Li H., Chen X., Peng X., Chen Y. (2021). Main active components of Jiawei Gegen Qinlian decoction protects against ulcerative colitis under different dietary environments in a gut microbiota-dependent manner. Pharmacol. Res..

[9] Yang Q., Vijayakumar A., Kahn B.B. (2018). Metabolites as regulators of insulin sensitivity and metabolism. Nat. Rev. Mol. Cell Biol..

[10] Urasaki Y., Pizzorno G., Le T.T. (2016). Chronic Uridine Administration Induces Fatty Liver and Pre-Diabetic Conditions in Mice. PLoS ONE.

[11] Zheng Y., Liang C., Li Z., Chen J., Chen Z., Jiang Y., Dong Q., Xiao Y., Fu C., Liao W. (2022). Study on the mechanism of Huangqin Decoction on rats with ulcerative colitis of damp-heat type base on mtDNA, TLR4, p-PI3K, p-Akt protein expression and microbiota. J. Ethnopharmacol..

[12] Suqin Y. (2023). Role Mechanism of Fecal Microbiota Transplantation on the Intervention of Ulcerative Colitis of Dampness-heat Syndrome Based on the Bitter-cold Medicinal Properties of Chinese Medicine Jinzhi. Chin. Med. Mod. Distance Educ. China.

[13] Okayasu I., Hatakeyama S., Yamada M., Ohkusa T., Inagaki Y., Nakaya R. (1990). A novel method in the induction of reliable experimental acute and chronic ulcerative colitis in mice. Gastroenterology.

[14] Low D., Nguyen D.D., Mizoguchi E. (2013). Animal models of ulcerative colitis and their application in drug research. Drug Des. Dev. Ther..

[15] David L.A., Maurice C.F., Carmody R.N., Gootenberg B.D., Button J.E., Wolfe B.E., Ling A.V., Devlin A.S., Varma Y., Fischbach M.A. (2014). Diet rapidly and reproducibly alters the human gut microbiome. Nature.

[16] Le Berre C., Honap S., Peyrin-Biroulet L. (2023). Ulcerative colitis. Lancet.

[17] Mo X., Tang K., Deng L., Zhou X., Li X., Zhang Y., Wang J. (2022). Prevention of ulcerative colitis by Huangqin decoction: Reducing the intestinal epithelial cell apoptosis rate through the IFN-γ/JAK/ETS signalling pathway. Pharm. Biol..

[18] Sun Y., Yuan S., Chen X., Sun J., Kalla R., Yu L., Wang L., Zhou X., Kong X., Hesketh T. (2023). The Contribution of Genetic Risk and Lifestyle Factors in the Development of Adult-Onset Inflammatory Bowel Disease: A Prospective Cohort Study. Am. J. Gastroenterol..

[19] Piovani D., Danese S., Peyrin-Biroulet L., Nikolopoulos G.K., Lytras T., Bonovas S. (2019). Environmental Risk Factors for Inflammatory Bowel Diseases: An Umbrella Review of Meta-analyses. Gastroenterology.

[20] Bolte L.A., Vich Vila A., Imhann F., Collij V., Gacesa R., Peters V., Wijmenga C., Kurilshikov A., Campmans-Kuijpers M.J.E., Fu J. (2021). Long-term dietary patterns are associated with pro-inflammatory and anti-inflammatory features of the gut microbiome. Gut.

[21] Hou J.K., Abraham B., El-Serag H. (2011). Dietary intake and risk of developing inflammatory bowel disease: A systematic review of the literature. Am. J. Gastroenterol..

[22] Wang C., Chu Q., Dong W., Wang X., Zhao W., Dai X., Liu W., Wang B., Liu T., Zhong W. (2024). Microbial metabolite deoxycholic acid-mediated ferroptosis exacerbates high-fat diet-induced colonic inflammation. Mol. Metab..

[23] Gu Q., Xia C., Liu N., Chen Z., Zhou Q., Li P. (2023). Lactobacillus plantarum ZJ316 alleviates ulcerative colitis by inhibiting inflammation and regulating short-chain fatty acid levels and the gut microbiota in a mouse model. Food Funct..

[24] Wu A., Gao Y., Kan R., Ren P., Xue C., Kong B., Tang Q. (2023). Alginate Oligosaccharides Prevent Dextran-Sulfate-Sodium-Induced Ulcerative Colitis via Enhancing Intestinal Barrier Function and Modulating Gut Microbiota. Foods.

[25] Bastaki S.M., Adeghate E., Amir N., Ojha S., Oz M. (2018). Menthol inhibits oxidative stress and inflammation in acetic acid-induced colitis in rat colonic mucosa. Am. J. Transl. Res..

[26] Yuan Z., Yang L., Zhang X., Ji P., Hua Y., Wei Y. (2019). Huang-Lian-Jie-Du Decoction Ameliorates Acute Ulcerative Colitis in Mice via Regulating NF-κB and Nrf2 Signaling Pathways and Enhancing Intestinal Barrier Function. Front. Pharmacol..

[27] Cui L., Guan X., Ding W., Luo Y., Wang W., Bu W., Song J., Tan X., Sun E., Ning Q. (2021). *Scutellaria baicalensis* Georgi polysaccharide ameliorates DSS-induced ulcerative colitis by improving intestinal barrier function and modulating gut microbiota. Int. J. Biol. Macromol..

[28] Wei X., Li N., Wu X., Cao G., Qiao H., Wang J., Hao R. (2023). The preventive effect of Glycyrrhiza polysaccharide on lipopolysaccharide-induced acute colitis in mice by modulating gut microbial communities. Int. J. Biol. Macromol..

[29] Chen Z., Dai G., Wu X., Li L., Tian Y., Tan L. (2023). Protective effects of Fagopyrum dibotrys on oxidized oil-induced oxidative stress, intestinal barrier impairment, and altered cecal microbiota in broiler chickens. Poult. Sci..

[30] Zhang Q., Gao X., Wu J., Chen M. (2022). The Correlation between Endotoxin, D-Lactate, and Diamine Oxidase with Endoscopic Activity in Inflammatory Bowel Disease. Dis. Markers.

[31] Gu W., Zhang L., Han T., Huang H., Chen J. (2022). Dynamic Changes in Gut Microbiome of Ulcerative Colitis: Initial Study from Animal Model. J. Inflamm. Res..

[32] Wu B., Zhou Q., He Z., Wang X., Sun X., Chen Y. (2021). Protective Effect of the *Abelmoschus manihot* Flower Extract on DSS-Induced Ulcerative Colitis in Mice. Evid. Based Complement. Alternat. Med..

[33] Zamani M., Alizadeh-Tabari S., Singh S., Loomba R. (2022). Meta-analysis: Prevalence of, and risk factors for, non-alcoholic fatty liver disease in patients with inflammatory bowel disease. Aliment. Pharmacol. Ther..

[34] Xu D., Ma R., Ju Y., Song X., Niu B., Hong W., Wang R., Yang Q., Zhao Z., Zhang Y. (2022). Cholesterol sulfate alleviates ulcerative colitis by promoting cholesterol biosynthesis in colonic epithelial cells. Nat. Commun..

