# Supplementary material for: High-Fat/High-Sugar Diet and High-Temperature/High-Humidity Exposure Aggravates Ulcerative Colitis in an Experimental Mouse Model
Source: Curr Issues Mol Biol. 2025 Jul 18;47(7):562. doi: 10.3390/cimb47070562 (PMC12293846; doi:10.3390/cimb47070562)
Supplement: Supplementary file 1 [file cimb-47-00562-s001.zip › cimb-3727521-supplementary.pdf]

Supplementary Information for

High-fat/high-sugar diet and high-temperature/high-humidity exposure aggravates ulcerative colitis in an experimental mouse model

Pengyan Li [1,†], Guibing Meng [1,†], Ang Li [1], Liang Chen [2], Xinchi Feng [1,*,‡], and Feng Qiu [1,*,‡]

1 School of Chinese Materia Medica, Tianjin Key Laboratory of Therapeutic Substance of Traditional and State Key Laboratory of Component-based Chinese Medicine, Tianjin University of Traditional Chinese Medicine, Tianjin China; pengyanlpy@163.com (P.L.); mgb901029@163.com (G.M.); lianghbkd@163.com (A.L.); xiaochi0211@163.com (X.F.); fengqiu20070118@163.com (F.Q.)

2 School of Pharmacy, Guizhou University of Traditional Chinese Medicine, Guiyang 550025, China; chenliang029@gzy.edu.cn (L.C.)

* Correspondence: xiaochi0211@163.com (X.F.); fengqiu20070118@163.com (F.Q.); Tel.: +86-22-595-6223 (F.Q.)

† Contributed equally to this work.

‡ Co-senior authorship.

Table S1. Disease activity index (DAI) score.

Weight loss (%)	Stool property	Blood stool	DAI score
<1	Normal	Negative of occult blood	0
1~5	Mild soft stool	Occult blood weakly positive (+)	1
5~10	Severe soft stool	Occult blood positive (++)	2
10~20	Mild diarrhea	Blood to the naked eye	3
>20	Severe diarrhea	Worse and blood naked eye	4

Table S2. Primer sequences.

Gene	Forward (5′-3′)	Reverser (5′-3′)
IL-1β	TGCCACCTTTTGACAGTGATG	TGTGCTGCTGCGAGATTTGA
IL-6	GTCCTTCCTACCCCAATTTCCA	TAACGCACTAGGTTTGCCGA
IL-17A	ACTACCTCAACCGTTCCACG	TTCCCTCCGCATTGACACAG
IFN-γ	GCCATCGGCTGACCTAGAGA	AGCCAGAAACAGCCATGAG
β-actin	GGCTGTATTCCCCTCCATCG	CCAGTTGGTAACAATGCCATGT

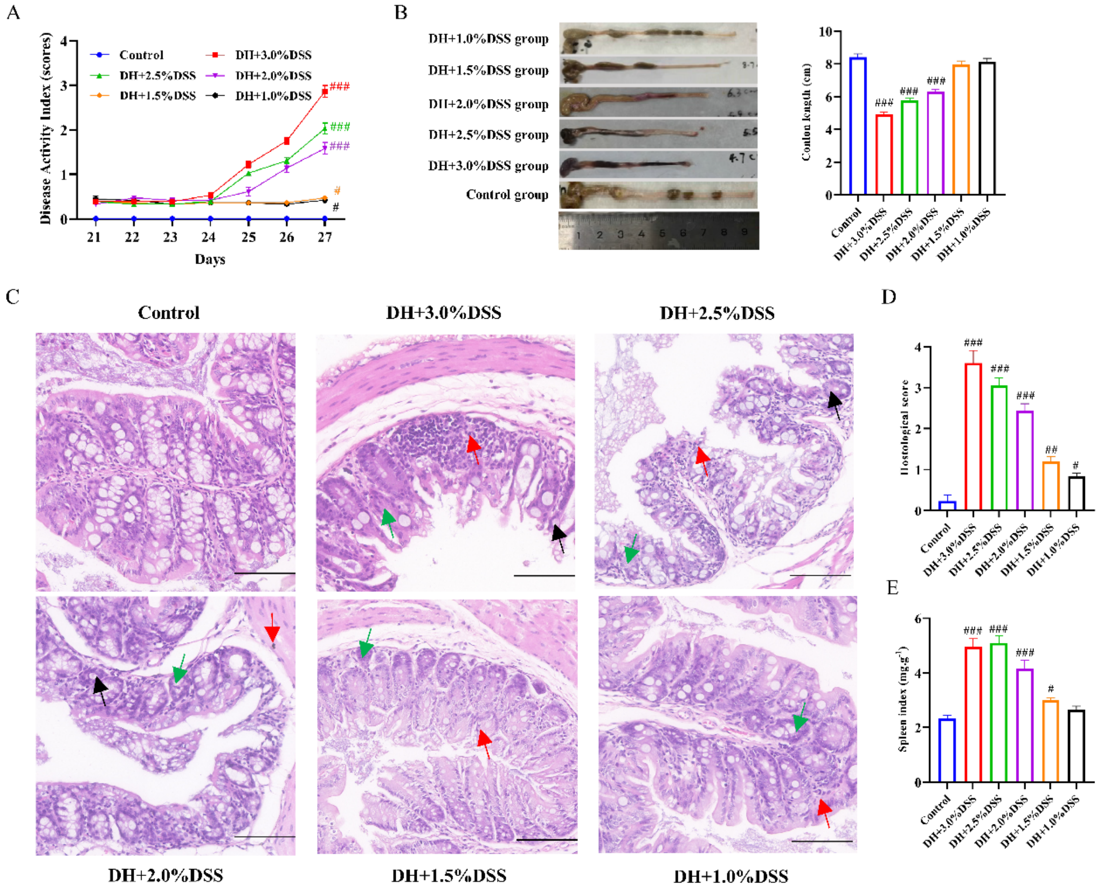

Figure S1. General conditions of mice in different groups. (A) Disease Activity Index (DAI) scores; (B) Representative pictures of colon tissues and colon length; (C) Representative results hematoxylin and eosin (H&E) staining. Red arrows indicate inflammatory cell infiltration; Black arrows indicate crypt abscess; Green arrows indicate loss of crypt structure and depletion of goblet cells. (D) Pathological lesion score of colonic tissues in each group; (E) Spleen index. Scale bar, 200 μm. All data are expressed as the mean ± SEM. $^{\#}p < 0.05$, $^{\#\#}p < 0.01$, $^{\#\#\#}p < 0.001$, $^{\#\#\#\#}p < 0.0001$ vs the control group.

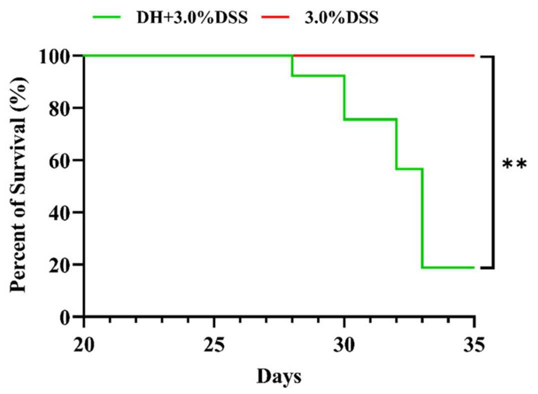

Figure S2. Survival curves of each group. Data are expressed as the mean ± SEM. $^{**}p < 0.01$, compared with 2.0% DSS group.
